# Supplementary material for: Monolithic three-dimensional integration of RRAM-based hybrid memory architecture for one-shot learning
Source: Nat Commun. 2023 Nov 6;14:7140. doi: 10.1038/s41467-023-42981-1 (PMC10628152; doi:10.1038/s41467-023-42981-1)
Supplement: Supplementary file 1 — Supplementary Information [file 41467_2023_42981_MOESM1_ESM.pdf]

Supplementary Information

Monolithic three-dimensional integration of RRAM-based hybrid memory architecture for one-shot learning

Yijun Li[1], Jianshi Tang[1,2,*], Bin Gao[1,2], Jian Yao[3], Anjunyi Fan[4,5], Bonan Yan[4,5], Yuchao Yang[4,5,6,7], Yue Xi[1], Yuankun Li[1], Jiaming Li[1], Wen Sun[1], Yiwei Du[1], Zhengwu Liu[1], Qingtian Zhang[1,2], Song Qiu[3], Qingwen Li[3], He Qian[1,2], and Huaqiang Wu[1,2*]

[1]School of Integrated Circuits, Tsinghua University, Beijing, China;

[2]Beijing Innovation Center for Future Chips, BNRist, Tsinghua University, Beijing, China;

[3]Suzhou Institute of Nano-Tech and Nano-Bionics, Chinese Academy of Science, Suzhou, China

[4]Institute for Artificial Intelligence, Peking University, Beijing, China

[5]Beijing Advanced Innovation Center for Integrated Circuits, School of Integrated Circuits, Peking University, Beijing, China

[6]School of Electronic and Computer Engineering, Peking University, Shenzhen, China

[7]Center for Brain Inspired Intelligence, Chinese Institute for Brain Research (CIBR), Beijing, China

[*]Corresponding e-mail: jtang@tsinghua.edu.cn; wuhq@tsinghua.edu.cn

Table of Contents

Supplementary Note 1: Detailed experimental method of results shown in Figure 2i-k............**2**

Supplementary Note 2: Detailed experimental method of results shown in Figure 3c. **4**

Supplementary Note 3: Testing method for Supplementary Fig.8. ....**6**

Supplementary Note 4: Evaluation method for the energy consumption of the CIM array.......**7**

Supplementary Fig. 1. Overall experimental setup of the M3D-LIME.....................................**9**

Supplementary Fig. 2. Method used for programming an analog 1T1R cell **10**

Supplementary Fig. 3. Array-level endurance and retention results (CIM layer) **11**

Supplementary Fig.4. Characterization of CNTFETs (TCAM layer) **12**

Supplementary Fig. 5. Statistical results of CNTFETs (TCAM layer) **13**

Supplementary Fig. 6. Characterizations of 1T1R half-cell (TCAM layer).............................**14**

Supplementary Fig. 7. Waveforms of pulse set and reset 1T1R buffer cell (TCAM layer).....**15**

Supplementary Fig. 8. Characterizations of 2T2R TCAM cell (TCAM layer)........................**16**

Supplementary Fig. 9. The τ and τ^{-1} extracted from the waveform in Figure 3c **17**

Supplementary Fig. 10. Structure of the MANN for one-shot learning **18**

Supplementary Fig. 11. Architecture illustration of the 2D chip... ....**19**

Supplementary Fig. 12. Illustration of the pipeline implementation of MANN **20**

Supplementary Fig. 13. Illustration of a scale-up M3D-LIME chip. **21**

Supplementary Table 1. Illustration of storing and searching 1-bit data with a 2T2R cell in the TCAM layer **23**

Supplementary Table 2. Key parameters of the MANN implemented in this work for one-shot learning...**24**

Supplementary Table 3. Execution time benchmark..**25**

Supplementary Table 4. Energy efficiency benchmark..**27**

Supplementary Note 1: Experimental method of results shown in Figure 2i-k.

In this experiment, we employed a multi-channel memory testing system in conjunction with the peripheral circuitry integrated on-chip with the CIM array (as depicted in Supplementary Fig.1) to demonstrate the calculation of MVM. The steps are summarized below:

To begin, we first mapped the weight matrix into the CIM array by programming. Initially, we determined the mapping relationship between the weights and the RRAM conductance values. For this demonstration, we set the memory window of the RRAM in the range of 0.4 to 40 μS, divided evenly into 16 conductance states (equivalent to 4 bits). Each weight in the weight matrix was quantized to 4 bits and then mapped to the respective RRAM conductance state, serving as the target value for programming the CIM array.

Subsequently, using the test system, we programmed each RRAM in the CIM array based on its target conductance using the standard write-verify method. We input a 7-bit binary address through the WL address input port of the CIM array to select the WL of the RRAM to be programmed. By controlling the voltage applied to the gate of the transistor connected to the selected WL through the WL reference voltage port, while keeping other WLs at a constant voltage of 0 V to ensure the connected transistors were turned off and prevent cross-talk, we could select one row of RRAMs (a total of 8) via WLs and then select the individual RRAMs one by one using the BLs (Bit Lines) orthogonal to the WLs.

For programming each 1T1R cell as shown in Supplementary Fig.2, we defined an interval based on the target conductance and the programming margin. The objective was to program the RRAM conductance within this interval, for which we applied a series of set and reset pulses. After each pulse, we read the conductance of the RRAM. If it exceeded the upper limit of the interval, we performed a reset operation in the next cycle. Conversely, if it fell below the lower limit, we performed a set operation in the next cycle. This process continued until the RRAM conductance was within the interval or the maximum number of cycles was reached, indicating the completion of

programming. We then proceeded to program the next RRAM. The set and reset pulses had a width of 50 ns, and their voltages ranged from 1 V to 3 V, with WL reference voltages of 1 V to 3 V for set pulses and 5 V for reset pulses. For reading the conductance, we applied a WL reference voltage of 5 V and a BL voltage of 0.15 V.

Once all the RRAMs were programmed, we completed the mapping of the weight matrix to the CIM array. By controlling the WL address input and BL, we read the results of the entire programmed array, as depicted in Figure 2i. The difference between this result and the target conductance value is shown in Figure 2j.

After that, we proceeded to input a series of vectors into the CIM array to demonstrate the MVM calculation. These input vectors followed a Gaussian distribution with a mean of 0 and a variance of 1, with each element of the vectors representing the elements of the matrix. These vectors were multiplied by the target conductance matrix, yielding the MVM result vector, where each element represented the expected inner product output.

Subsequently, by controlling the WL address and BL voltage, we performed 100 read operations on the CIM array. These operations were subject to read noise and write errors, resulting in fluctuating conductance matrices compared to the target conductance matrix. We multiplied the input vectors with these matrices, obtaining the predicted MVM results from the CIM array. In order to compare the difference between the predicted MVM results and the theoretical results, we considered the predicted inner product output as a function of its theoretical value, i.e., the expected inner product output. To mitigate the influence of output amplitude on the results, the data was normalized as plotted in Figure 2k, which demonstrates the capability of the CIM array in performing the MVM calculation.

Supplementary Note 2: Experimental method of results shown in Figure 3c.

Instruments utilized for this experiment included the Keysight B1500 and B1530 Semiconductor Analyzers, Keysight B2201 Switch Matrix, Tektronix MS054 Oscilloscope, and Probe Card System. To demonstrate the calculation of Hamming distance, we followed the steps outlined below:

Firstly, for each 2T2R cell in the TCAM arrays, we programmed all the RRAMs according to the scheme described in Supplementary Table 1. Specifically, we sequentially programmed each RRAM device by applying suitable voltages to the SELs, while turning off the driving transistors of the unselected RRAM (typical gate voltage of 0 V) and setting the gate voltage of the transistor driving the selected RRAM (typical gate voltage of -2.5 V). Next, we applied appropriate operating voltages to the ML and SL for DC programming of the devices. For the set operation, we applied 0V to the ML and -2 V to the SL. For the reset operation, we applied -3 V to the ML and 0V to the SL. After the set/reset operation, we then performed DC readout of the programmed RRAMs by applying -0.15 V to the ML, 0 V to the SL, -5 V to the SEL of the selected RRAM, and 0V to the SEL of the unselected RRAMs. If the resistance of the selected RRAM did not meet the requirements (typical values were around 30 kΩ for LRS and above 5 MΩ for HRS), we then applied a series of 50 ns pulses to continue programming the RRAM. After each 50 ns pulse operation, we read the resistance value of the RRAM. If it did not meet the requirements, we increased the pulse operating voltage for the next cycle until the resistance value fell within the target range to complete the programming. We repeated this process for each device until every unit in the 5×1 TCAM array was correctly programmed. Through these operations, we successfully stored the template vector in the TCAM array. For the testing in Figure 3c, a '00000' vector is stored.

After that, search vectors were inputted through the SELs to calculate the Hamming distance. In this experiment, we first connected the ML to the oscilloscope and set the source electrode of the pre-charging transistor to 1 V. Then, based on each bit of the search vector, we defined the on and off states of each transistor during the search

process according to the scheme outlined in Supplementary Table 1. For the transistors in the off state, their connected SELs were grounded with SL, resulting in a gate voltage of 0 throughout the search process. For the SELs of transistors in the on state during the search process and the gate electrode of the pre-charging transistor, they were respectively connected to two pulse sources. Initially, the pre-charging transistor was turned on (typically with a gate voltage of -4 V) to charge the ML. After a certain period of time (typically 1 ms), the pre-charging transistor was turned off (typically with a gate voltage of 1 V), followed by a delay (typically 1 μs). Then, by applying a -5 V pulse to the corresponding SELs, we turned on the corresponding transistors and triggered the oscilloscope to record the discharge waveform of the ML. The relevant pulses were applied through a set of PMUs of Keysight B1530, enabling good synchronization of the signals. By implementing search vectors with different Hamming distances from the stored template and recording the results on the waveforms, we obtained the results depicted in Figure 3c.

Supplementary Note 3: Testing method for Supplementary Fig.8.

We built a testing system with Keysight B1500 and B1530 for TCAM-related testing. Initially, we programmed the 2T2R TCAM cells using the configuration detailed in Supplementary Table 1. This involved applying distinct voltages to the gate terminals of the two CNTFETs to turn on the one driving the selected RRAM while keeping the other one driving the unselected RRAM turned off (V_{GS}=0). We also applied the appropriate voltages to the ML and SL in order to program each RRAM in the 2T2R cell. The typical voltages for set operation were V_{GS}=-3 V and V_{ML}=-2 V, whereas V_{GS}=-5 V and V_{SL}=-3 V for reset. Following the programming of the 2T2R cells, we conducted the 1-bit search operation on them using the configuration outlined in Supplementary Table 1. This included applying a voltage of 0.15 V to the ML and appropriate V_{GS} voltages to the two CNTFETs, then reading the current and calculating the resistance. In cases where the search data did not match the stored template, the CNTFET driving the RRAM in LRS opened and the CNTFET driving the RRAM in HRS closed, leading to a lower resistance known as mismatch resistance. Conversely, if the search data matched the stored template, the CNTFET driving the HRS opened and the CNTFET driving the LRS closed, resulting in a larger resistance known as match resistance. The ratio between the match and mismatch resistances serves as a key parameter for RRAM-based TCAM as it determines the length of the WL, or the search length.

Supplementary Note 4: Evaluation method for the energy consumption of the CIM array (as presented in Supplementary Table 4.b).

In this work, we employed the XPESim simulator, an open-source platform (available at https://github.com/thuime/XPEsim) introduced by our previous work (Wenqiang Zhang, et al., DAC, 2019, doi: 10.1145/3316781.3317797), to assess the energy consumption of the CIM array. Here the energy consumption of the CIM array was evaluated as follows:

First, the input parameters for the XPESim simulator were determined. Our measurement results of the CIM array indicated that the RRAM conductance was in the range of 4-40 μS. For the specific task, four CIM array sizes were employed: 9×64, 9×128, 6217×8, and 8×128. Also, 130 nm technology node was used here.

Using these parameters, the power consumption calculations were performed using XPESim. This evaluation can be divided into three components:

1) The power consumption of the RRAM array was primarily determined by the array size and RRAM conductance range, which were taken as input parameters, while the weight distribution was simulated using XPESim. For instance, an 8×128 array with a conductance range of 4-40 μS resulted in a power consumption value of 32.6 pJ for the RRAM array.
2) The power consumption of the peripheral circuitry and drivers, including DACs, WL/SL switches and MUX, was mainly determined by the technology node as well as the number of inputs and outputs of the CIM array. XPESim utilized DC synthesis and layout analysis to calculate the power consumption based on these parameters. For example, in an 8×128 array with a technology node of 130 nm, the power consumption values were estimated to be 8.7 pJ for the WL switch, 3.3 pJ for the SL switch, and 11.1 pJ for the MUX.
3) The power consumption of ADCs was mainly determined by the technology node and the number of outputs. XPESim referred to the appropriate references using the technology node of 130 nm (e.g., 5.04-fJ/conversion-Step: Taimur Rabuske, et al., IEEE TVLSI, 2014, doi: 10.1109/TVLSI.2014.2337236) and calculated the

ADC's power consumption based on the number of outputs. For instance, for an array with 128 outputs, the power consumption was found to be 0.17 nJ: $\mathbf{5.04\ (fJ/conversion - setp) \times 2^{8\,(bits)} \times 128\ (outputs) = 0.17\ nJ}$.

Finally, we summed up the power consumption of peripheral circuitry, drivers, and ADCs and recorded it under the label of "CMOS" in Supplementary Table 4b.

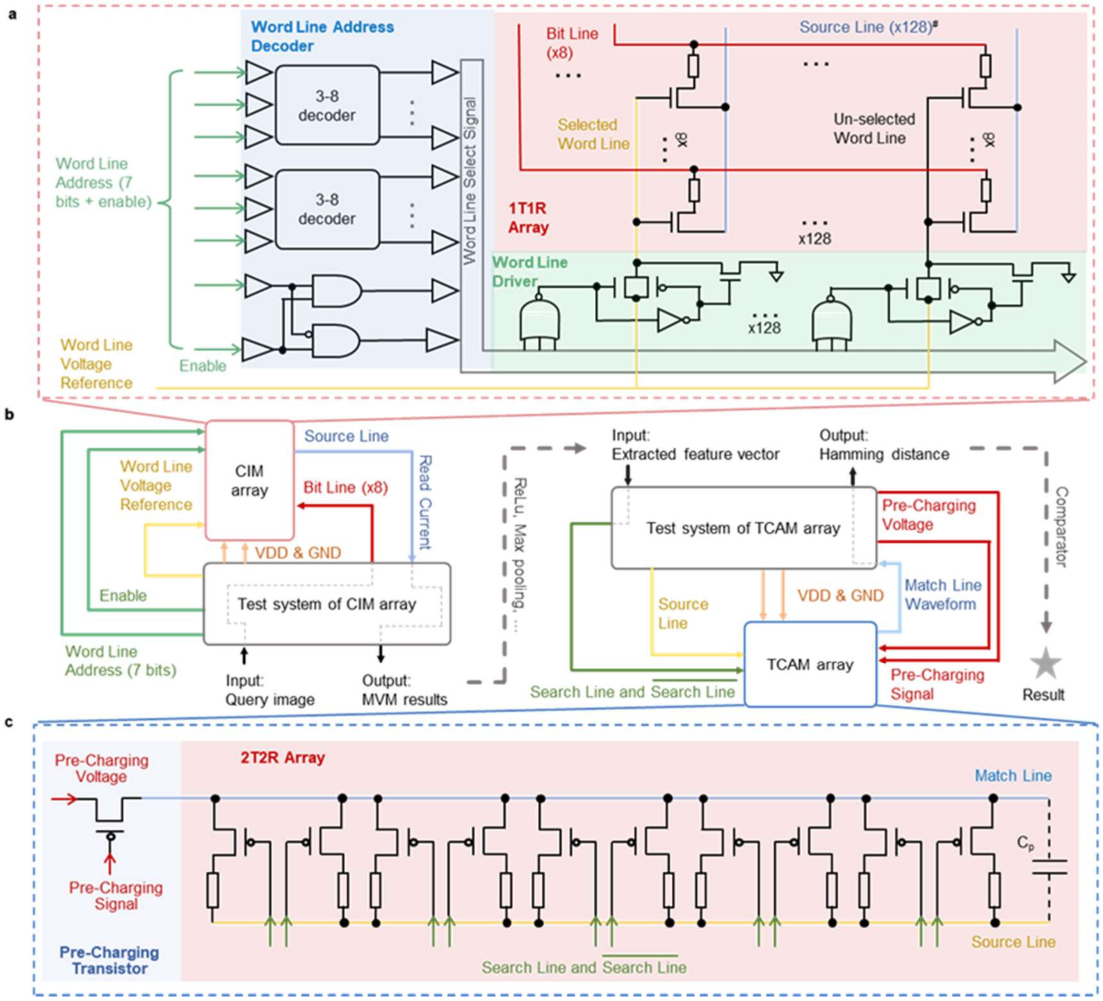

Supplementary Fig. 1. Overall experimental setup of the M3D-LIME. (**a**) Schematic diagram of the 1Kb CIM array used for implementing MVM calculations. The peripheral circuits are implemented using Si CMOS logic in the first layer of M3D-LIME chip. #Usually it is preferred not to connect all the SLs together in order to perform fully parallel MVM calculation. However, in practical circuit design, multiple or all the SLs could be connected together to share one ADC, which helps save the hardware cost as well as reduce the number of test pads and simplify testing. In this case, the selector transistors of a specific row can be activated through the WLs, allowing the MVM calculation on one SL at one time. This enables sequential MVM calculation. (**b**) Illustration of the signal transmissions between the CIM array, TCAM array and the testing system. (**c**) Schematic diagram of the 5×1 TCAM array used for implementing Hamming distance calculations. The CNTFETs and Ta_2O_5-based RRAMs of the TCAM array are fabricated in the 3rd layer.

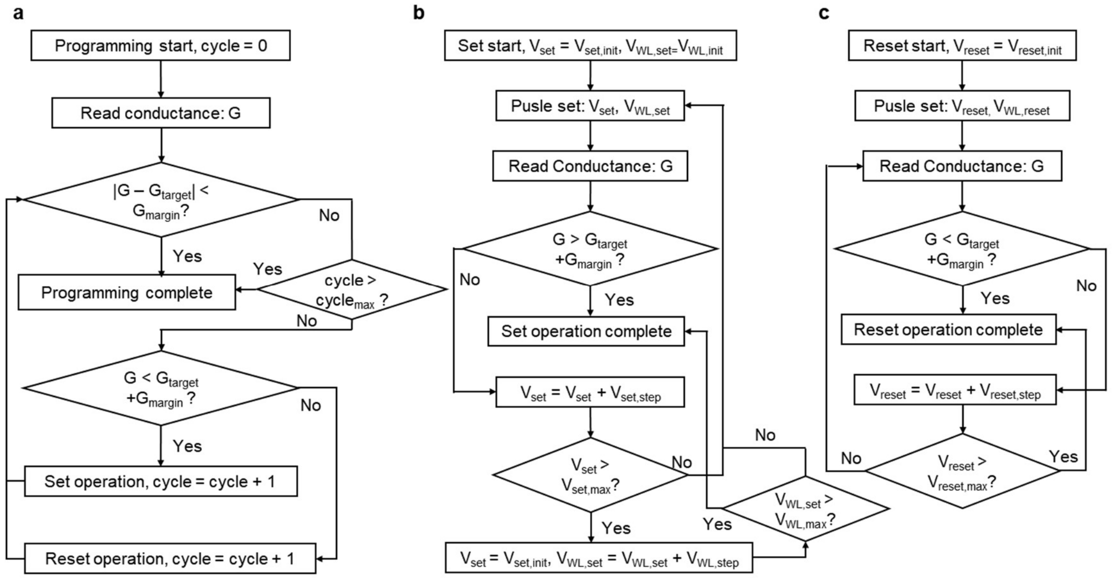

Supplementary Fig.2. Programming of analog 1T1R cells in the 2nd layer of CIM array. (**a**) Illustration of the overall programming process. (**b**) Illustration of the set operation. (**c**) Illustration of the reset operation.

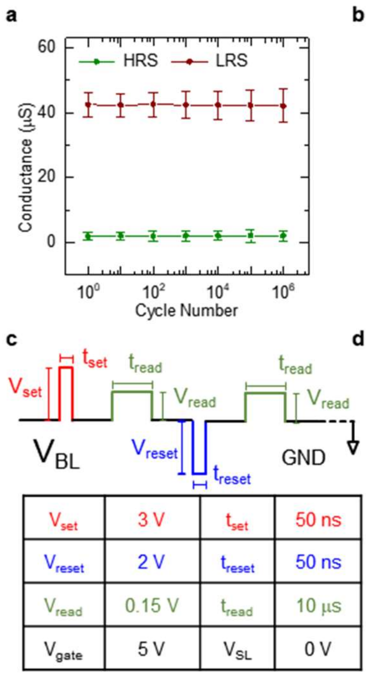

V_{set}	3 V	t_{set}	50 ns
V_{reset}	2 V	t_{reset}	50 ns
V_{read}	0.15 V	t_{read}	10 μs
V_{gate}	5 V	V_{SL}	0 V

Supplementary Fig. 3. Array-level endurance and retention measurement on the analog RRAM-based CIM array. (**a**) Endurance test of the CIM array with 1024 1T1R cells. In each cycle, the 1T1R cells were mapped to the LRS and HRS using a write-verify scheme. The read voltage was 0.2 V. (**b**) Retention test of 8 representative conductance states. For the retention test, 1T1R cells in the 1k-bit array were mapped to 8 representative conductance states with 128 cells for each state. After mapping, their conductance was continuously measured after every second, and the average conductance of 128 1T1R cells for each state is plotted. (**c**) Illustration of full-fledged endurance test using pulse operation and reading (Mario Lanza, et al., Adv. Elec. Mater., 2019, doi:10.1002/aelm.201800143). The waveform of bit line voltage is plotted with the key parameters listed in the table below. (**d**) Full-fledged endurance test result of a representative 1T1R cell.

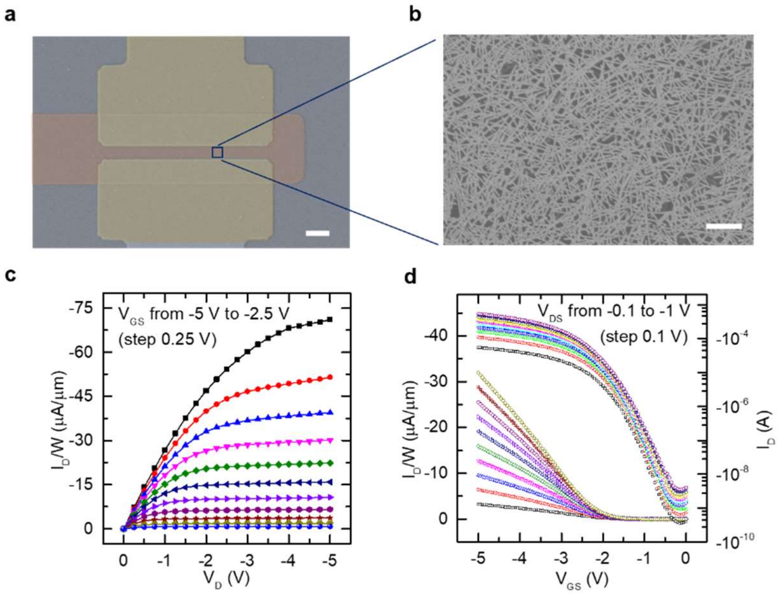

Supplementary Fig. 4. Characterization of CNTFET in the TCAM layer. (**a**) False-color SEM image of a back-gated CNTFET. Scale bar: 4 μm. The designed channel width and length are 20 μm and 2 μm, respectively, while the gate dielectric is 10 nm-thick HfO_2. (**b**) SEM image of the wet-transferred CNT film in the channel of CNTFET. Scale bar: 1 μm. (**c**) I_D-V_D output curves of a typical CNTFET, with gate voltage varying from -5 to -1 V. (**d**) I_D-V_{GS} transfer curves of a typical CNTFET, with drain voltage varying from -0.1 to -1 V.

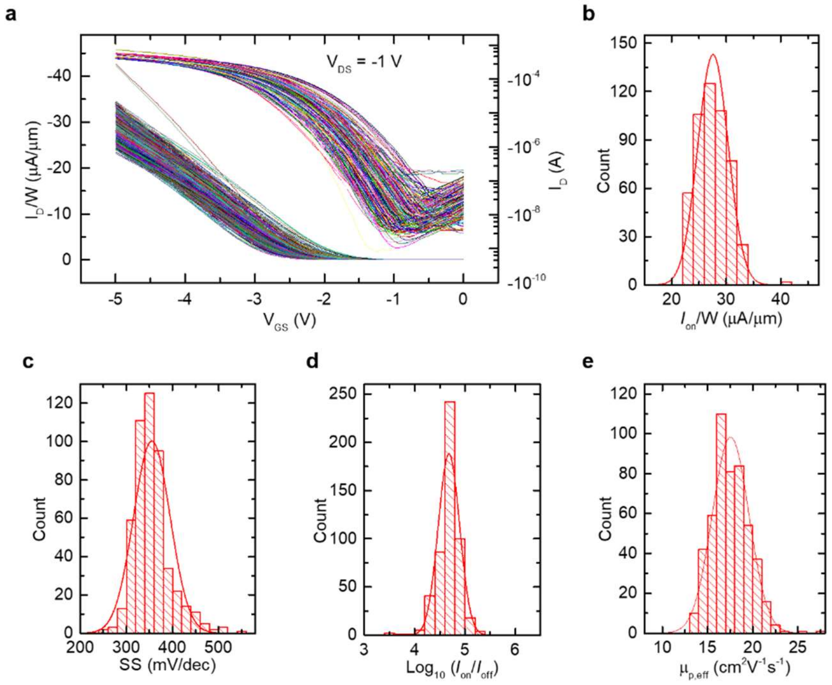

Supplementary Fig. 5. Statistical results of 500 CNTFETs. (**a**) I_D-V_{GS} transfer curves of 500 measured CNTFETs with a drain voltage of -1 V. Histograms of (**b**) On-state current density, (**c**) subthreshold swing, (**d**) on/off ratio, and (**e**) field-effect carrier mobility. The CNTFETs have a channel size of W/L=20 μm/2 μm.

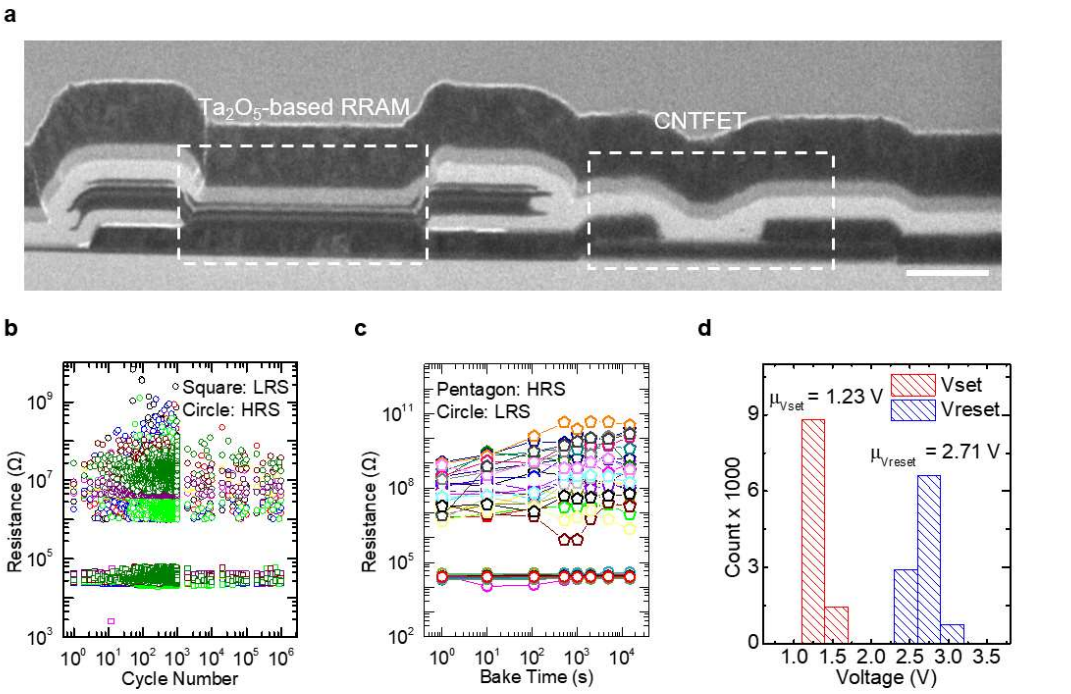

Supplementary Fig. 6. Characterizations of 1T1R half-cell in the TCAM layer. (**a**) Cross-sectional TEM image of a 1T1R half-cell, scale bar: 200 nm. (**b**) Endurance test and (**c**) retention test (baking at 120 ºC) of a typical 1T1R half-cell, exhibiting a large HRS/LRS ratio. (**d**) Histogram of set and reset voltages during the endurance test mentioned in (b). The endurance test involved the measurement on 10 1T1R half-cells, while the retention test was performed on 25 1T1R half-cells with LRS and 25 1T1R half-cells with HRS. The CNTFETs used to drive the Ta_2O_5-based RRAM cells have a channel size of W/L=20 μm/2 μm.

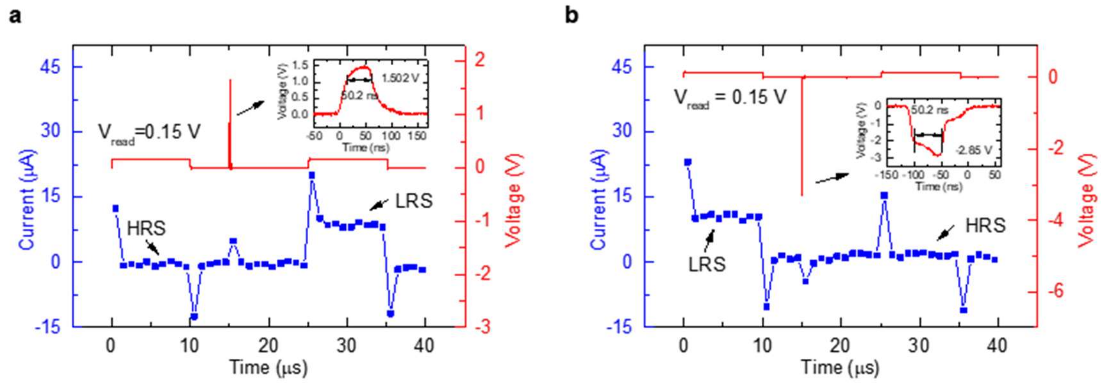

Supplementary Fig.7. Waveforms of set/reset pulse operations on 1T1R cell in the TCAM layer. (**a**) Waveform of the BL voltage and current during the set operation. The pulse width is ~50ns. (**b**) Waveform of the BL voltage and current during the reset operation. The pulse width is also ~50ns. Initially, a 0.15V pulse was used to measure the initial resistance (current) of the RRAM. Subsequently, a 50 ns pulse with a set voltage of 1.5V or a reset voltage of 3V was applied to operate the RRAM. Finally, a 0.15V pulse was used to measure the post-operation resistance (current) of the RRAM. The results demonstrated that RRAM can be successfully programmed by a 50 ns pulse, with a set voltage of 1.5 V and a reset voltage of 3 V.

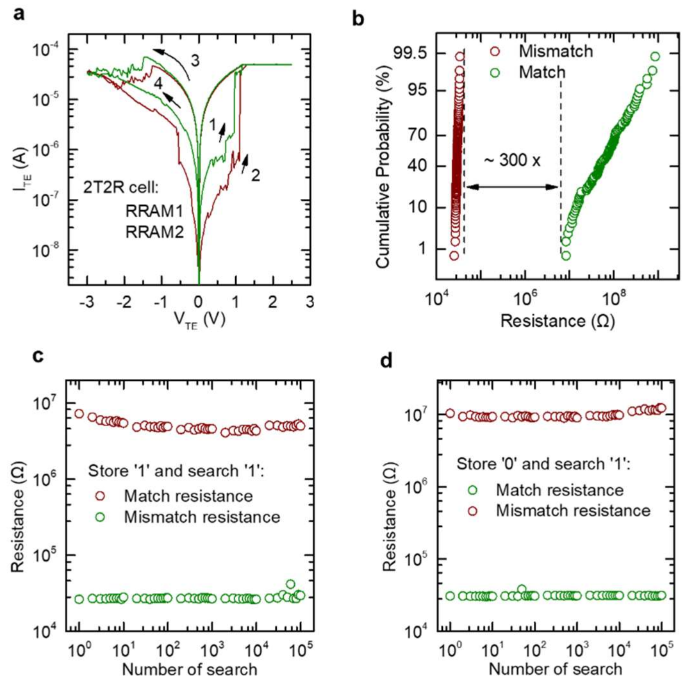

Supplementary Fig. 8. Characterizations of 2T2R TCAM cells in the TCAM layer. (**a**) DC I-V curves of two 1T1R half-cells in a 2T2R TCAM cell. (**b**) Distribution of discharging resistance for 100 2T2R cells, showing a large match/mismatch resistance ratio >300×. Results of the read disturb test are shown in (c) and (d): (**c**) Search '1' when '1' is stored in the TCAM cell. (**d**) Search '1' when '0' is stored in the TCAM cell. In the search operation, 100-ns pulses with a voltage of 1 V are applied to the TE (ML). The CNTFETs used in the 2T2R TCAM cells have a channel size of 20 μm/2 μm.

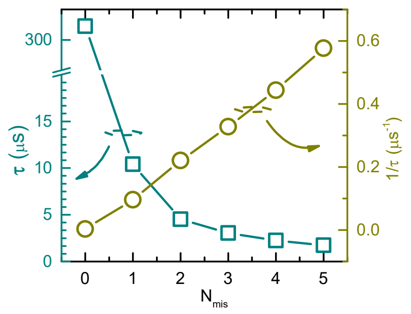

Supplementary Fig. 9. The calculated discharging time constant τ (left axis) and τ^{-1} (right axis) extracted from the waveform shown in Figure 3c in the main text.

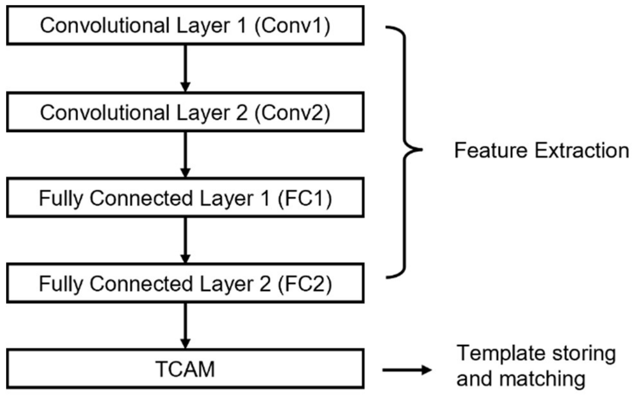

Supplementary Fig. 10. Structure of the MANN implemented in this work for one-shot learning.

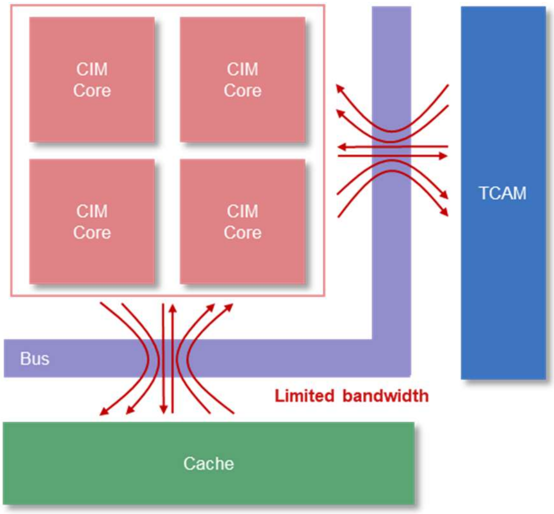

Supplementary Fig. 11. Architecture of the 2D baseline chip. In the 2D chip architecture, the CIM core, the TCAM, and the cache are realized in the same manner as in the M3D-LIME. However, the key difference lies in their way of on-chip data transfer, as the M3D-LIME uses high-bandwidth ILVs while the 2D counterpart employs a bus. Specifically, the CIM core is realized by $HfAlO_x$-based analog RRAM array and Si CMOS control circuits, while the TCAM and cache employ CNTFETs and Ta_2O_5-based binary RRAMs. In the benchmark evaluation, the bus has a bandwidth of 128 bits and a frequency of 200 MHz. Due to the limited bandwidth of the bus, substantial data transfer latency is incurred when performing parallel computing in multiple CIM arrays, which acts as a bottleneck for the overall system performance.

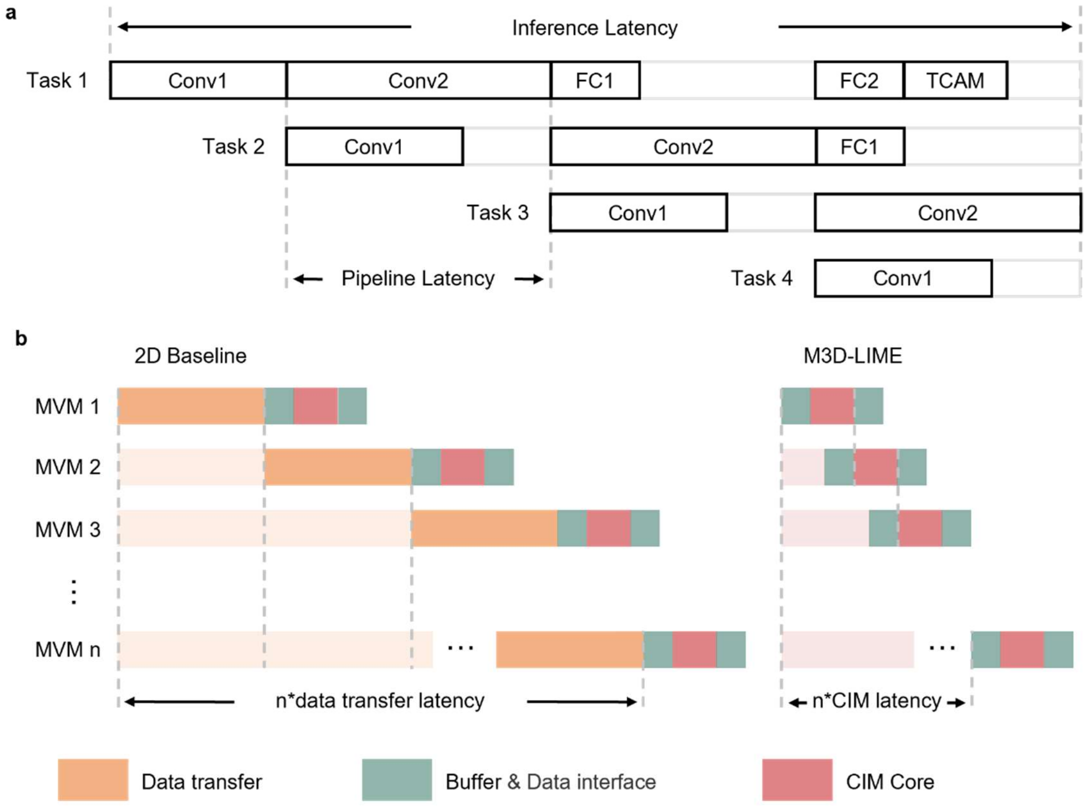

Supplementary Fig. 12. Illustration of the pipeline implementation of MANN. (**a**) Illustration of the inter-layer pipeline for the entire network. Inference tasks 1-4 are carried out in pipelined execution, and the computation in different layers is executed in parallel. (**b**) Illustration of the intra-layer pipeline for the implementation of each layer. Each layer is split into pipelined MVM operations composed of data transfer, CIM core, data transfer and buffer & data interface. In the M3D-LIME, the latency of each layer is significantly reduced by saving the data transfer cost.

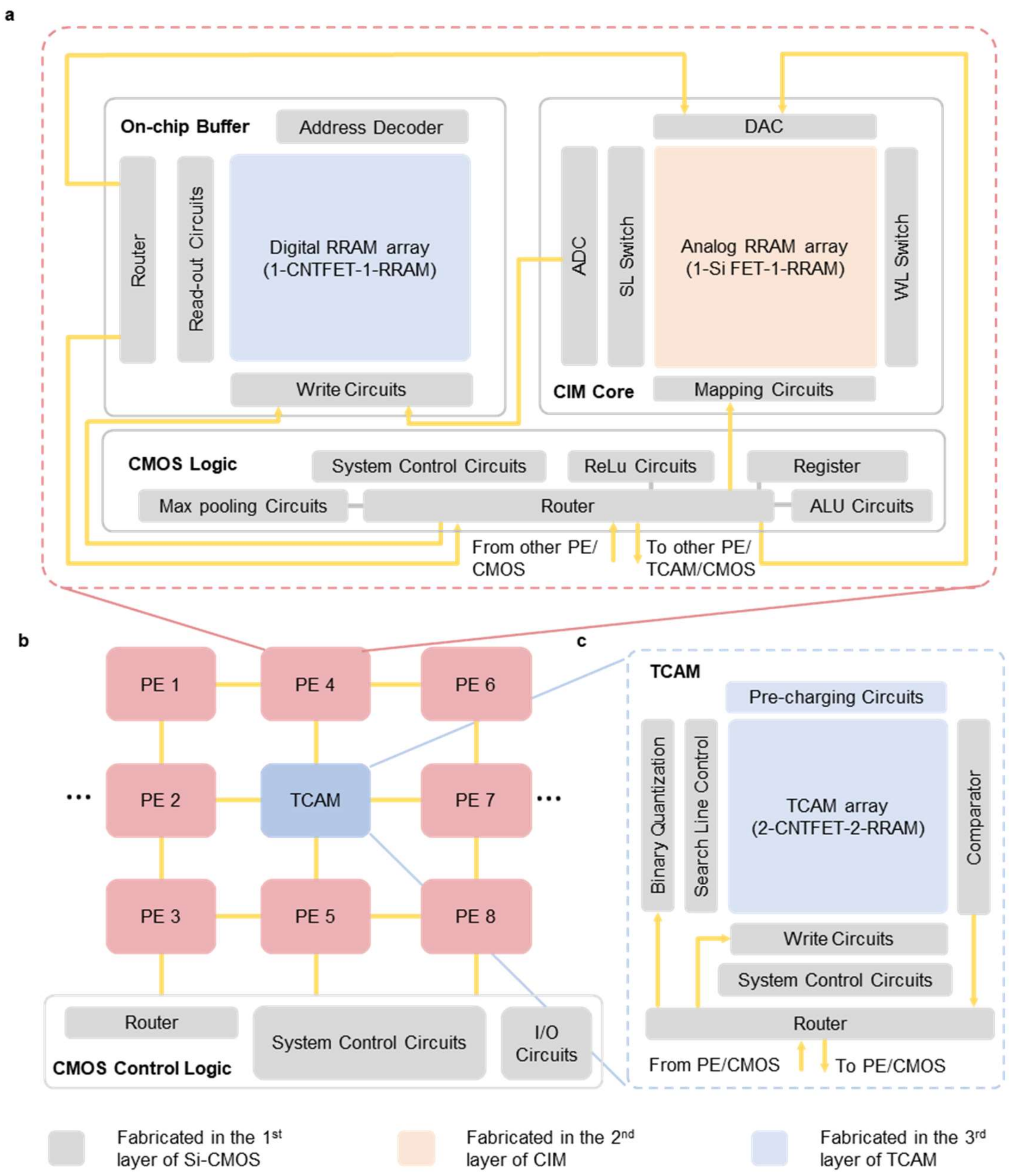

Supplementary Fig. 13. Illustration of a scale-up M3D-LIME chip. (**a**) Illustration of a processing unit (PE) that contains one CIM core, associated on-chip buffer and CMOS logic. (**b**) Illustration of a tile, containing multiple PEs, one TCAM and one CMOS control logic. (**c**) Illustration of a TCAM. The scale-up M3D-LIME chip could monolithically integrate multiple CIM arrays, the associated on-chip buffers, and one or more TCAM arrays, on top of Si CMOS logic circuits to efficiently implement large-scale MANNs. Each core represents a processing element (PE) that contains one CIM array, associated on-chip buffer and CMOS logic. Multiple PEs, one TCAM and one CMOS control logic could further form a tile. The number of PEs and tiles integrated

on the chip depends on the specific application as well as other factors such as the technology node and chip area. In addition to the on-chip buffers, network-on-chip (NoC) based on routers can be employed to facilitate communication between those cores.

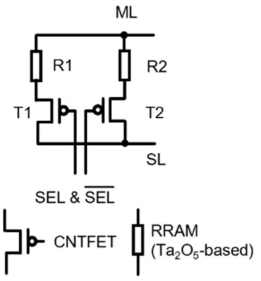

Store bit	Search bit	Discharge resistance of ML
'0' : R1-LRS R2-HRS	Search '1': T1-on, T2-off	Mismatch: LRS + $R_{T\text{-}on}$ // HRS + $R_{T\text{-}off}$ ≈ LRS
	Search '0': T1-off, T2-on	Match: LRS + $R_{T\text{-}off}$ // HRS + $R_{T\text{-}on}$ ≈ HRS
'1' : R1-HRS R2-LRS	Search '1': T1-on, T2-off	Match: HRS + $R_{T\text{-}on}$ // LRS + $R_{T\text{-}off}$ ≈ HRS
	Search '0': T1-off, T2-on	Mismatch: HRS + $R_{T\text{-}off}$ // LRS + $R_{T\text{-}on}$ ≈ LRS
'X': R1-HRS R2-HRS	Search '1': T1-on, T2-off	HRS + $R_{T\text{-}on}$ // HRS + $R_{T\text{-}off}$ ≈ HRS
	Search '0': T1-off, T2-on	HRS + $R_{T\text{-}off}$ // HRS + $R_{T\text{-}on}$ ≈ HRS

Supplementary Table 1. Illustration of storing and searching 1-bit data with a 2T2R cell in the TCAM layer.

Convolutional Layer 1	
Conv	1×64×3×3
BN	-
ReLU	-
MaxPooling	2×2
Dropout	-

Convolutional Layer 2	
Conv	64×128×3×3
BN	-
ReLU	-
MaxPooling	2×2
Dropout	-

Fully Connected Layers and TCAM	
FC1	6272×8
FC2	8×128
Binary Quantization	-
TCAM	128×5

Supplementary Table 2. Key parameters of the MANN implemented in this work for one-shot learning.

a

Parameters of Execution Time Benchmark		
	M3D-LIME	2D Baseline
Technology Node	130 nm	
Bandwidth of Bus	-	128 bit
Clock	200 MHz	200 MHz
Size of the Input Image	28 * 28	
Buffer write latency	50 ns	
Buffer read latency	10 ns	
CIM core latency*	50 ns	
Binary Quantization latency	5 ns	
TCAM latency	100 ns#	

b

Execution Time Benchmark&				
		Array number	Array size	Latency
Conv1	M3D-LIME	4	9 * 64	9860 ns
	2D Baseline			11758.4 ns
Conv2	M3D-LIME	64	9 * 128	9860 ns
	2D Baseline			26920 ns
FC1	M3D-LIME	1	6217 * 8	110 ns
	2D Baseline			2070 ns
FC2	M3D-LIME	1	8 * 128	110 ns
	2D Baseline			115 ns
TCAM	M3D-LIME	1	5 * 128	115 ns
	2D Baseline			
Average / pipeline latency of M3D-LIME				9860 ns
Average / pipeline latency of 2D Baseline				26920 ns

Supplementary Table 3. Parameters (a) and results (b) of the benchmark of execution time. *The CIM core latency includes the time from when the data is first inputted to when the computation is completed and the final output is provided by the ADCs. #The TCAM latency of 100ns for a search length of 128 is roughly extrapolated from the experimental data in Figure 3c (where the discharging time for N_{mis} = 5 with a search length of 5 is about 3 μs) using the formula: $\tau = 3\ \mu s/128\times5=117$ ns. We shall point out that the measured discharging time constant is mainly limited by the parasitic capacitance in our testing system (including a switching matrix and probe card). The TCAM search time can be significantly reduced if we eliminate the undesired parasitic

capacitance and also shrink the channel length of CNTFET that helps reduce the ML pre-charging time. [&]The max pooling time is taken into consideration, but it actually does not increase the overall computation time, since it can be implemented using high-speed digital logic circuits and performed in parallel with the MVM calculation in the next stage.

a

Energy Consumption of TCAM	
R_{match} / $R_{mismatch}$	30 kΩ / 200 MΩ (1 bit)
V_{ML}	1 V
Average Search Energy	[(1 V)^2 / 30 kΩ + (1 V)^2 / 200 MΩ] / 2 * 100 ns / bit = 1.67 pJ/bit

b

Energy Consumption of CIM Arrays[#]				
	9 * 64	9 * 128	6217 * 8	8 * 128
CMOS[&]	91.3 pJ	0.17 nJ	7.85 nJ	0.17 nJ
CIM array	18.3 pJ	36.7 pJ	7.36 nJ	32.6 pJ
Total	0.11 nJ	0.21 nJ	15.2 nJ	0.20 nJ

c

Energy Consumption of Buffer	
LRS / HRS	30 kΩ / 200 MΩ
Write Voltage	3 V (Reset) / 1.5 V (Set)
Average Write Energy	[(3 V)^2 / 30 kΩ + (1.5 V)^2 / 200 MΩ] / 2 * 50 ns / bit = 7.50 pJ/bit
Read Voltage	0.15 V
Average Read Energy	[(0.15 V)^2 / 30 kΩ + (0.15 V)^2 / 200 MΩ] / 2 * 10 ns / bit = 3.75 fJ/bit

d

Energy Consumption of M3D-LIME	
Conv1	0.11 nJ * 28 * 28 + (64 + 9) * (3.75 fJ/bit+ 7.50 pJ/bit) * 28 * 28 = 0.52 μJ[*]
Conv2	0.21 nJ * 64 * 14 * 14 + (128 + 9) * (3.75 fJ/bit+ 7.50 pJ/bit) * 64 * 14 * 14 = 15.5 μJ
FC1	15.2 nJ + (6217 + 8) * (3.75 fJ/bit+ 7.50 pJ/bit) = 61.9 nJ
FC2	0.20 nJ + (128 + 8) * (3.75 fJ/bit+ 7.50 pJ/bit) = 1.22 nJ
TCAM	1.67 pJ/bit * 5 * 128 = 1.07 nJ
Total	16.1 μJ

e

Energy Efficiency Comparison		
	M3D-LIME	GPU[##]
Average Latency	9860 ns	-
Energy Consumption	16.3 μJ	-
Energy Efficiency	29.9 MOP / 16.3 μJ = 1.83 TOPs^{-1}W^{-1}	30.4 TOPs^{-1} / 300 W = 0.1 TOPs^{-1}W^{-1}

Supplementary Table 4. Energy efficiency benchmark. Energy consumption evaluation for (**a**) TCAM, (**b**) CIM arrays, (**c**) buffer and (**d**) M3D-LIME as well as (**e**) comparison of energy efficiency with GPU. [#]These values are obtained by the simulator XPEsim (Wenqiang Zhang, et al., DAC, 2019, doi: 10.1145/3316781.3317797). [&]Peripheral CMOS circuits, including the ADCs, DACs, WL switch, and BL switch are all taken into consideration. [*]Input image size is 28 × 28. [##]The reported energy

efficiency value of the Nvidia Tesla V100 GPU (Stefano Ambrogio, et al., Nature, 2018, doi: 10.1038/s41586-018-0180-5), which has been extensively utilized as a standard reference in numerous prior studies is included for comparison in the energy efficiency benchmark against GPUs. The Nvidia Tesla V100 GPU is fabricated at 12 nm technology node.
